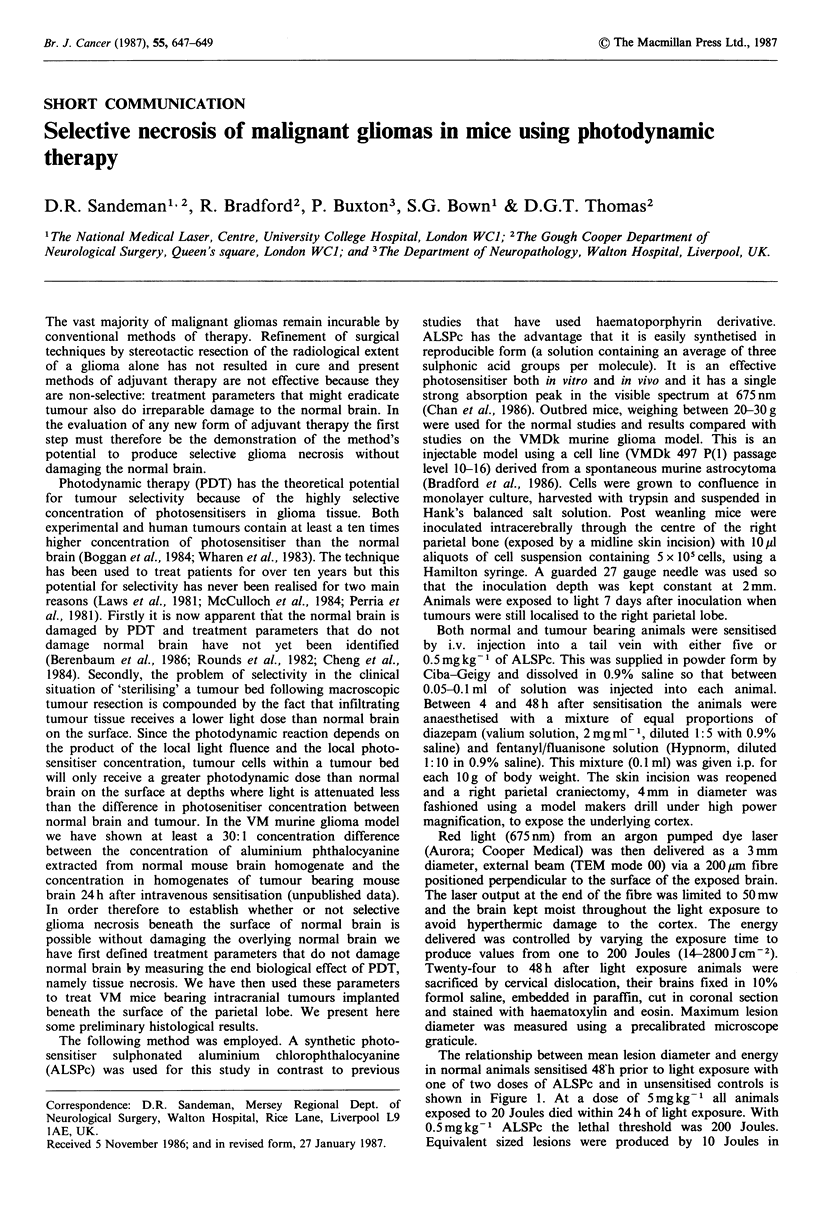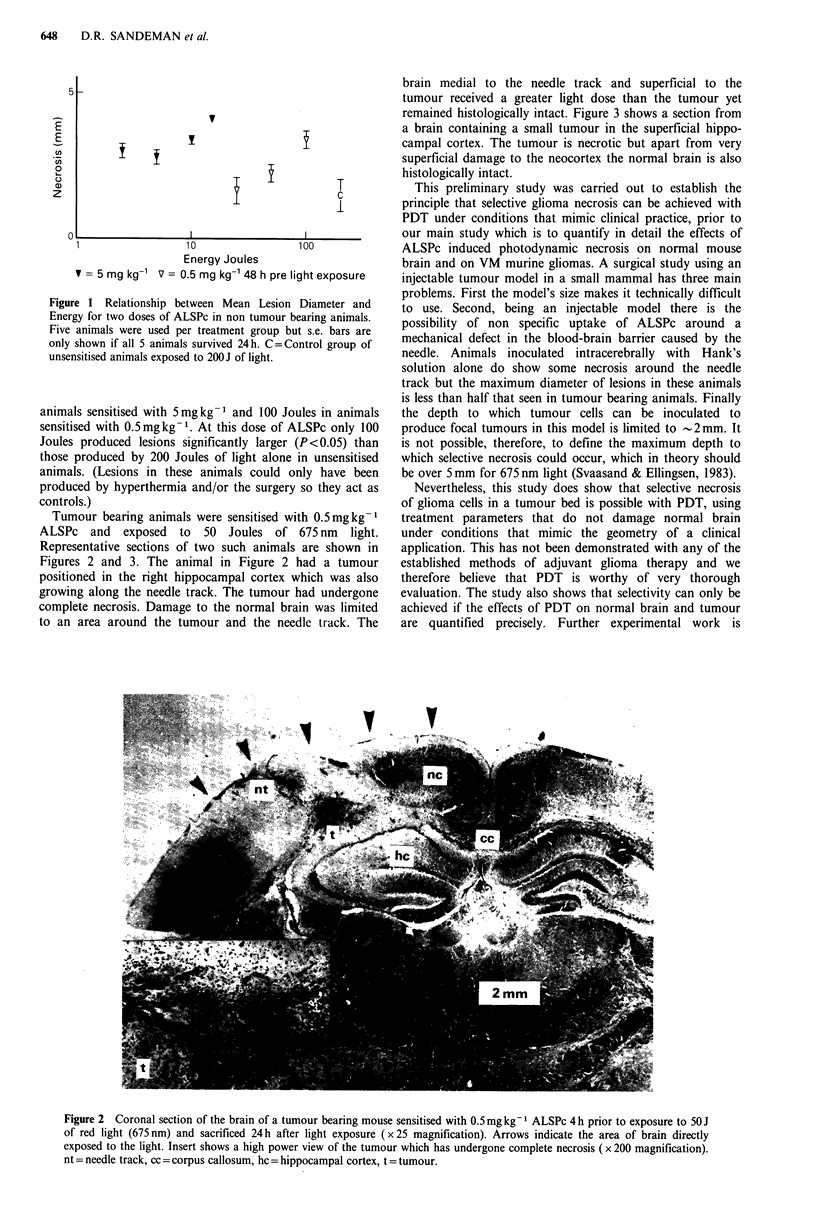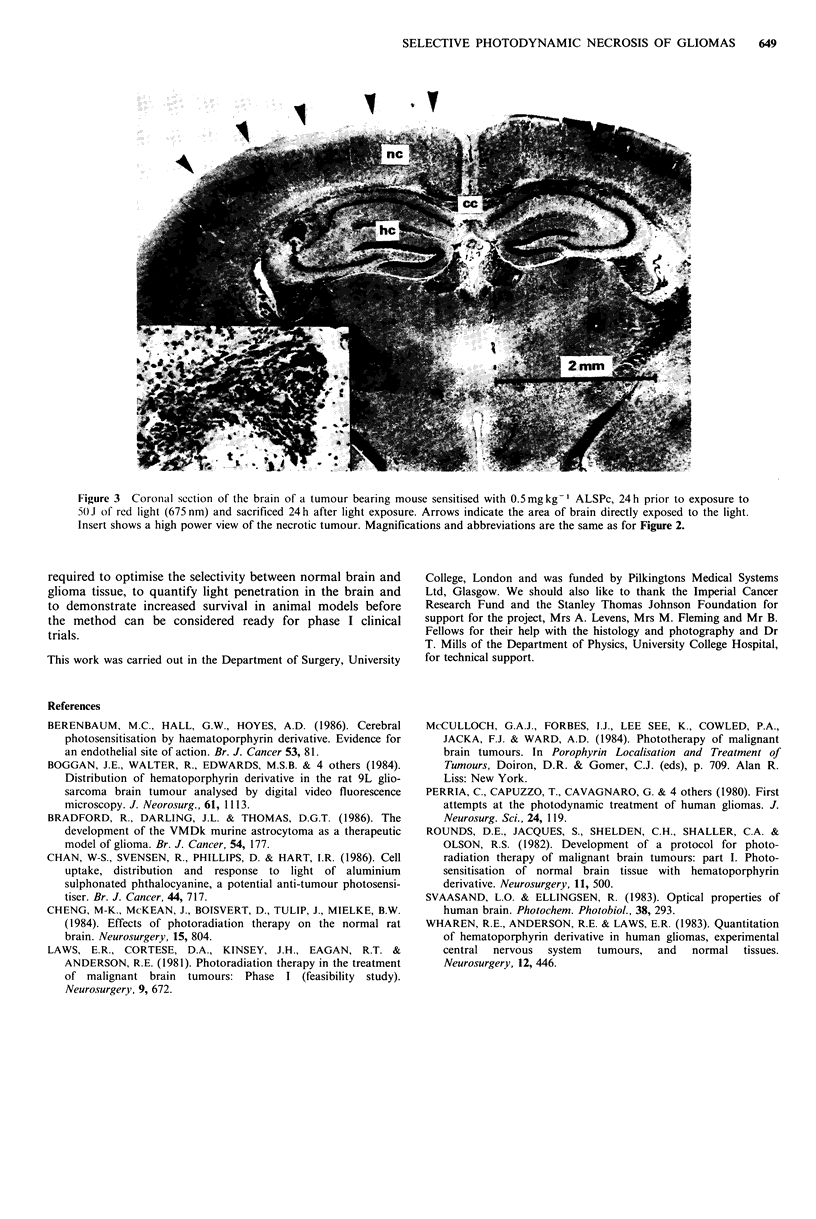# Selective necrosis of malignant gliomas in mice using photodynamic therapy.

**DOI:** 10.1038/bjc.1987.131

**Published:** 1987-06

**Authors:** D. R. Sandeman, R. Bradford, P. Buxton, S. G. Bown, D. G. Thomas

## Abstract

**Images:**


*Br. J. Cancer* (1987), **55**, 647–649 © The Macmillan Press Ltd., 1987

SHORT COMMUNICATION

### Selective necrosis of malignant gliomas in mice using photodynamic therapy

D.R. Sandeman[1,2], R. Bradford[2], P. Buxton[3], S.G. Bown[1] & D.G.T. Thomas[2]

[1]*The National Medical Laser, Centre, University College Hospital, London WC1;* [2]*The Gough Cooper Department of Neurological Surgery, Queen's square, London WC1; and* [3]*The Department of Neuropathology, Walton Hospital, Liverpool, UK.*

The vast majority of malignant gliomas remain incurable by conventional methods of therapy. Refinement of surgical techniques by stereotactic resection of the radiological extent of a glioma alone has not resulted in cure and present methods of adjuvant therapy are not effective because they are non-selective: treatment parameters that might eradicate tumour also do irreparable damage to the normal brain. In the evaluation of any new form of adjuvant therapy the first step must therefore be the demonstration of the method's potential to produce selective glioma necrosis without damaging the normal brain.

Photodynamic therapy (PDT) has the theoretical potential for tumour selectivity because of the highly selective concentration of photosensitisers in glioma tissue. Both experimental and human tumours contain at least a ten times higher concentration of photosensitiser than the normal brain (Boggan *et al.*, 1984; Wharen *et al.*, 1983). The technique has been used to treat patients for over ten years but this potential for selectivity has never been realised for two main reasons (Laws *et al.*, 1981; McCulloch *et al.*, 1984; Perria *et al.*, 1981). Firstly it is now apparent that the normal brain is damaged by PDT and treatment parameters that do not damage normal brain have not yet been identified (Berenbaum *et al.*, 1986; Rounds *et al.*, 1982; Cheng *et al.*, 1984). Secondly, the problem of selectivity in the clinical situation of 'sterilising' a tumour bed following macroscopic tumour resection is compounded by the fact that infiltrating tumour tissue receives a lower light dose than normal brain on the surface. Since the photodynamic reaction depends on the product of the local light fluence and the local photosensitiser concentration, tumour cells within a tumour bed will only receive a greater photodynamic dose than normal brain on the surface at depths where light is attenuated less than the difference in photosenitiser concentration between normal brain and tumour. In the VM murine glioma model we have shown at least a 30:1 concentration difference between the concentration of aluminium phthalocyanine extracted from normal mouse brain homogenate and the concentration in homogenates of tumour bearing mouse brain 24 h after intravenous sensitisation (unpublished data). In order therefore to establish whether or not selective glioma necrosis beneath the surface of normal brain is possible without damaging the overlying normal brain we have first defined treatment parameters that do not damage normal brain by measuring the end biological effect of PDT, namely tissue necrosis. We have then used these parameters to treat VM mice bearing intracranial tumours implanted beneath the surface of the parietal lobe. We present here some preliminary histological results.

The following method was employed. A synthetic photosensitiser sulphonated aluminium chlorophthalocyanine (ALSPc) was used for this study in contrast to previous studies that have used haematoporphyrin derivative. ALSPc has the advantage that it is easily synthetised in reproducible form (a solution containing an average of three sulphonic acid groups per molecule). It is an effective photosensitiser both *in vitro* and *in vivo* and it has a single strong absorption peak in the visible spectrum at 675 nm (Chan *et al.*, 1986). Outbred mice, weighing between 20–30 g were used for the normal studies and results compared with studies on the VMDk murine glioma model. This is an injectable model using a cell line (VMDk 497 P(1) passage level 10–16) derived from a spontaneous murine astrocytoma (Bradford *et al.*, 1986). Cells were grown to confluence in monolayer culture, harvested with trypsin and suspended in Hank's balanced salt solution. Post weanling mice were inoculated intracerebrally through the centre of the right parietal bone (exposed by a midline skin incision) with 10 $\mu$l aliquots of cell suspension containing $5\times10^5$ cells, using a Hamilton syringe. A guarded 27 gauge needle was used so that the inoculation depth was kept constant at 2 mm. Animals were exposed to light 7 days after inoculation when tumours were still localised to the right parietal lobe.

Both normal and tumour bearing animals were sensitised by i.v. injection into a tail vein with either five or 0.5 mg kg$^{-1}$ of ALSPc. This was supplied in powder form by Ciba–Geigy and dissolved in 0.9% saline so that between 0.05–0.1 ml of solution was injected into each animal. Between 4 and 48 h after sensitisation the animals were anaesthetised with a mixture of equal proportions of diazepam (valium solution, 2 mg ml$^{-1}$, diluted 1:5 with 0.9% saline) and fentanyl/fluanisone solution (Hypnorm, diluted 1:10 in 0.9% saline). This mixture (0.1 ml) was given i.p. for each 10 g of body weight. The skin incision was reopened and a right parietal craniectomy, 4 mm in diameter was fashioned using a model makers drill under high power magnification, to expose the underlying cortex.

Red light (675 nm) from an argon pumped dye laser (Aurora; Cooper Medical) was then delivered as a 3 mm diameter, external beam (TEM mode 00) via a 200 $\mu$m fibre positioned perpendicular to the surface of the exposed brain. The laser output at the end of the fibre was limited to 50 mw and the brain kept moist throughout the light exposure to avoid hyperthermic damage to the cortex. The energy delivered was controlled by varying the exposure time to produce values from one to 200 Joules (14–2800 J cm$^{-2}$). Twenty-four to 48 h after light exposure animals were sacrificed by cervical dislocation, their brains fixed in 10% formol saline, embedded in paraffin, cut in coronal section and stained with haematoxylin and eosin. Maximum lesion diameter was measured using a precalibrated microscope graticule.

The relationship between mean lesion diameter and energy in normal animals sensitised 48 h prior to light exposure with one of two doses of ALSPc and in unsensitised controls is shown in Figure 1. At a dose of 5 mg kg$^{-1}$ all animals exposed to 20 Joules died within 24 h of light exposure. With 0.5 mg kg$^{-1}$ ALSPc the lethal threshold was 200 Joules. Equivalent sized lesions were produced by 10 Joules in

Correspondence: D.R. Sandeman, Mersey Regional Dept. of Neurological Surgery, Walton Hospital, Rice Lane, Liverpool L9 1AE, UK.
Received 5 November 1986; and in revised form, 27 January 1987.

**Figure 1** Relationship between Mean Lesion Diameter and Energy for two doses of ALSPc in non tumour bearing animals. Five animals were used per treatment group but s.e. bars are only shown if all 5 animals survived 24h. C=Control group of unsensitised animals exposed to 200 J of light.

animals sensitised with 5 mg kg$^{-1}$ and 100 Joules in animals sensitised with 0.5 mg kg$^{-1}$. At this dose of ALSPc only 100 Joules produced lesions significantly larger ($P<0.05$) than those produced by 200 Joules of light alone in unsensitised animals. (Lesions in these animals could only have been produced by hyperthermia and/or the surgery so they act as controls.)

Tumour bearing animals were sensitised with 0.5 mg kg$^{-1}$ ALSPc and exposed to 50 Joules of 675 nm light. Representative sections of two such animals are shown in Figures 2 and 3. The animal in Figure 2 had a tumour positioned in the right hippocampal cortex which was also growing along the needle track. The tumour had undergone complete necrosis. Damage to the normal brain was limited to an area around the tumour and the needle track. The brain medial to the needle track and superficial to the tumour received a greater light dose than the tumour yet remained histologically intact. Figure 3 shows a section from a brain containing a small tumour in the superficial hippocampal cortex. The tumour is necrotic but apart from very superficial damage to the neocortex the normal brain is also histologically intact.

This preliminary study was carried out to establish the principle that selective glioma necrosis can be achieved with PDT under conditions that mimic clinical practice, prior to our main study which is to quantify in detail the effects of ALSPc induced photodynamic necrosis on normal mouse brain and on VM murine gliomas. A surgical study using an injectable tumour model in a small mammal has three main problems. First the model's size makes it technically difficult to use. Second, being an injectable model there is the possibility of non specific uptake of ALSPc around a mechanical defect in the blood-brain barrier caused by the needle. Animals inoculated intracerebrally with Hank's solution alone do show some necrosis around the needle track but the maximum diameter of lesions in these animals is less than half that seen in tumour bearing animals. Finally the depth to which tumour cells can be inoculated to produce focal tumours in this model is limited to ~2 mm. It is not possible, therefore, to define the maximum depth to which selective necrosis could occur, which in theory should be over 5 mm for 675 nm light (Svaasand & Ellingsen, 1983).

Nevertheless, this study does show that selective necrosis of glioma cells in a tumour bed is possible with PDT, using treatment parameters that do not damage normal brain under conditions that mimic the geometry of a clinical application. This has not been demonstrated with any of the established methods of adjuvant glioma therapy and we therefore believe that PDT is worthy of very thorough evaluation. The study also shows that selectivity can only be achieved if the effects of PDT on normal brain and tumour are quantified precisely. Further experimental work is

**Figure 2** Coronal section of the brain of a tumour bearing mouse sensitised with 0.5 mg kg$^{-1}$ ALSPc 4 h prior to exposure to 50 J of red light (675 nm) and sacrificed 24 h after light exposure (×25 magnification). Arrows indicate the area of brain directly exposed to the light. Insert shows a high power view of the tumour which has undergone complete necrosis (×200 magnification). nt=needle track, cc=corpus callosum, hc=hippocampal cortex, t=tumour.

Figure 3 Coronal section of the brain of a tumour bearing mouse sensitised with $0.5\,mg\,kg^{-1}$ ALSPc, 24 h prior to exposure to 50 J of red light (675 nm) and sacrificed 24 h after light exposure. Arrows indicate the area of brain directly exposed to the light. Insert shows a high power view of the necrotic tumour. Magnifications and abbreviations are the same as for **Figure 2.**

required to optimise the selectivity between normal brain and glioma tissue, to quantify light penetration in the brain and to demonstrate increased survival in animal models before the method can be considered ready for phase I clinical trials.

This work was carried out in the Department of Surgery, University College, London and was funded by Pilkingtons Medical Systems Ltd, Glasgow. We should also like to thank the Imperial Cancer Research Fund and the Stanley Thomas Johnson Foundation for support for the project, Mrs A. Levens, Mrs M. Fleming and Mr B. Fellows for their help with the histology and photography and Dr T. Mills of the Department of Physics, University College Hospital, for technical support.

### References

BERENBAUM, M.C., HALL, G.W., HOYES, A.D. (1986). Cerebral photosensitisation by haematoporphyrin derivative. Evidence for an endothelial site of action. *Br. J. Cancer* **53,** 81.

BOGGAN, J.E., WALTER, R., EDWARDS, M.S.B. & 4 others (1984). Distribution of hematoporphyrin derivative in the rat 9L gliosarcoma brain tumour analysed by digital video fluorescence microscopy. *J. Neorosurg.*, **61,** 1113.

BRADFORD, R., DARLING, J.L. & THOMAS, D.G.T. (1986). The development of the VMDk murine astrocytoma as a therapeutic model of glioma. *Br. J. Cancer*, **54,** 177.

CHAN, W-S., SVENSEN, R., PHILLIPS, D. & HART, I.R. (1986). Cell uptake, distribution and response to light of aluminium sulphonated phthalocyanine, a potential anti-tumour photosensitiser. *Br. J. Cancer*, **44,** 717.

CHENG, M-K., McKEAN, J., BOISVERT, D., TULIP, J., MIELKE, B.W. (1984). Effects of photoradiation therapy on the normal rat brain. *Neurosurgery*, **15,** 804.

LAWS, E.R., CORTESE, D.A., KINSEY, J.H., EAGAN, R.T. & ANDERSON, R.E. (1981). Photoradiation therapy in the treatment of malignant brain tumours: Phase I (feasibility study). *Neurosurgery*, **9,** 672.

McCULLOCH, G.A.J., FORBES, I.J., LEE SEE, K., COWLED, P.A., JACKA, F.J. & WARD, A.D. (1984). Phototherapy of malignant brain tumours. In *Porophyrin Localisation and Treatment of Tumours*, Doiron, D.R. & Gomer, C.J. (eds), p. 709. Alan R. Liss: New York.

PERRIA, C., CAPUZZO, T., CAVAGNARO, G. & 4 others (1980). First attempts at the photodynamic treatment of human gliomas. *J. Neurosurg. Sci.*, **24,** 119.

ROUNDS, D.E., JACQUES, S., SHELDEN, C.H., SHALLER, C.A. & OLSON, R.S. (1982). Development of a protocol for photoradiation therapy of malignant brain tumours: part I. Photosensitisation of normal brain tissue with hematoporphyrin derivative. *Neurosurgery*, **11,** 500.

SVAASAND, L.O. & ELLINGSEN, R. (1983). Optical properties of human brain. *Photochem. Photobiol.*, **38,** 293.

WHAREN, R.E., ANDERSON, R.E. & LAWS, E.R. (1983). Quantitation of hematoporphyrin derivative in human gliomas, experimental central nervous system tumours, and normal tissues. *Neurosurgery*, **12,** 446.